# Anti‐Swelling Hydrogel Combined With Nucleus Pulposus Cell Exosomes and Senolytic Drugs Efficiently Repair Intervertebral Disc Degeneration

**DOI:** 10.1002/advs.202513645

**Published:** 2025-09-16

**Authors:** Songfeng Chen, Hao Han, Yuhao Zhang, Longyu Li, Zhishuo Wang, Jiaming Zhang, Liang Han, Hongwei Kou, Guowei Shang, Chunfeng Shang, Zikuan Leng, Keya Mao, Chengwei Li, Lin Jin, Hongjian Liu

**Affiliations:** ^1^ The First Affiliated Hospital of Zhengzhou University Zhengzhou 45000 China; ^2^ Sanquan College of Xinxiang Medical University Xinxiang 453003 China; ^3^ Department of Orthopedics the Chinese PLA General Hospital Beijing 100039 China; ^4^ School of Agriculture Sciences Zhengzhou University Zhengzhou 450001 China; ^5^ Henan Key Laboratory of Intelligent Biomaterials and Medical Applications，International Joint Research Laboratory for Biomedical Nanomaterials of Henan Zhoukou Normal University Zhoukou 466001 China

**Keywords:** anti‐swelling hydrogel, intervertebral disc degeneration, mitochondrial function, nucleus pulposus cell exosomes, senolytics, nucleus pulposus cells

## Abstract

Intervertebral disc degeneration (IVDD) is a multifactorial pathology primarily driven by the senescence of nucleus pulposus cells (NPC), inflammatory microenvironment of extracellular matrix (ECM), and the resultant decline in NPCs viability. Conventional treatment strategies often fail to address these two interconnected factors simultaneously. To overcome this limitation, a bifunctional anti‐swelling hydrogel system encapsulating anti‐senescence drugs quercetin (Q) and dasatinib (D), as well as nucleus pulposus‐derived exosomes (NP‐Exo) is developed. This system is designed to clear senescent NPCs, regulate the inflammatory disc microenvironment, and enhance NPC activity, thereby significantly improving treatment efficacy. Mechanistically, this strategy helps preserve mitochondrial function, maintain mitochondrial membrane potential, and reduce excessive reactive oxygen species production, which collectively contribute to delaying cellular senescence and restoring disc homeostasis. Additionally, the anti‐swelling property of the hydrogel can alleviate structural displacement caused by swelling, further optimizing the stability and efficacy of the treatment. The biological efficacy of this system is validated in both rat and goat models. The experimental results demonstrated that this drug delivery system can effectively restore the integrity of the ECM, ultimately promoting the repair of IVDD. These findings highlight the platform's potential for IVDD treatment, offering a novel therapeutic strategy for IVDD repair.

## Graphical abstract

In this study, a bifunctional anti‐swelling hydrogel system is developed, which encapsulated anti‐senescence drugs quercetin, dasatinib, nucleus pulposus‐derived exosomes (NP‐Exo) to clear senescent nucleus pulposus cells, regulate the inflammatory disc microenvironment, and enhance nucleus pulposus cells activity, ultimately promoting the repair of intervertebral disc degeneration.

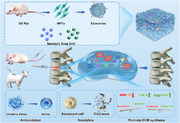

## 1 Introduction

Low back pain (LBP), primarily caused by intervertebral disc degeneration (IVDD), is a significant contributor to disability globally, imposing a substantial socioeconomic burden.^[^ ^1^ ^]^ IVDD is recognized as the pathological basis for many spinal degenerative diseases, such as disc herniation and spinal stenosis.^[^ ^2^ ^]^ The existing therapeutic paradigm for LBP, including both non‐surgical and surgical approaches, does not adequately address the central pathological mechanism of IVDD, which involves the intrinsic degenerative process of the intervertebral disc (IVD).^[^ ^3^, ^4^ ^]^ Therefore, there is a pressing need to explore the pathogenic mechanisms of IVDD and develop targeted therapeutic strategies.

Cellular senescence, particularly in nucleus pulposus cells (NPCs), is a major contributor to the progression of IVDD.^[^ ^5^, ^6^ ^]^ Senescent NPCs exhibit significant alterations in gene and protein expression, leading to impaired disc function and accelerated degeneration.^[^ ^7^, ^8^ ^]^ Additionally, during IVDD, notochordal cells in the nucleus pulposus region are gradually replaced by chondrocyte‐like cells, and proteoglycans are progressively lost, resulting in decreased water content of the nucleus pulposus, which further exacerbates cellular senescence and tissue degeneration.^[^ ^9^, ^10^, ^11^ ^]^ The accumulation of these senescent cells induces a pro‐inflammatory and oxidative microenvironment, creating a vicious cycle that exacerbates disc degeneration.^[^ ^12^ ^]^ This cycle is further compounded by cellular apoptosis and the failure of NPCs to maintain IVD homeostasis, which accelerates the progression of IVDD.^[^ ^5^ ^]^

Targeting this cycle of senescence, inflammation, and oxidative stress offers a promising strategy for repairing IVDD.^[^ ^13^, ^14^, ^15^ ^]^ Despite certain progress in this field, there are still significant limitations in the clearance of senescent cells, mainly reflected in the need for optimization of drug delivery strategies and insufficient drug targeting. Studies have shown that BCL‐2 inhibitors have good senolytic potential, but their off‐target effects limit clinical application.^[^ ^16^ ^]^ In addition, other potential anti‐aging targets, including the p53 pathway, anti‐apoptotic transcription factor FOXO4, deubiquitinase USP7^[^ ^17^ ^]^ and molecular chaperone HSP90,^[^ ^18^ ^]^ can promote the selective clearance of senescent cells through different mechanisms, but further optimization is still needed in terms of drug selectivity and toxicity control. In contrast, dasatinib (D) combined with quercetin (Q) as a classic senolytic agent, with high specificity and good safety, represents a breakthrough in this field. Previous studies have shown that the dasatinib and quercetin combination can not only selectively eliminate senescent cells but also effectively reduce the level of oxidative stress.^[^ ^19^ ^]^ These compounds have shown potential in mitigating inflammation and promoting cellular regeneration across various tissues. Therefore, we aim to investigate the effects of dasatinib and quercetin combination therapy on IVD repair, hypothesizing that this approach may restore NPCs function and slow the progression of IVDD. While clearing senescent cells can improve the disc microenvironment, this alone may not fully restore disc function. For optimal therapeutic efficacy, it is crucial to combine senescence clearance with strategies that enhance NPCs activity. This dual approach is vital for promoting IVD regeneration.

In addition to cellular therapy, exosomes derived from NPCs have emerged as a promising therapeutic tool.^[^ ^20^ ^]^ These exosomes carry various bioactive molecules—including miRNAs, proteins, and lipids—derived from their parent cells. They mediate intercellular communication, regulate local inflammation, inhibit apoptosis, promote cell survival, and participate in tissue repair. Compared to exosomes derived from mesenchymal stem cells, nucleus pulposus‐derived exosomes (NP‐Exo) demonstrate superior biocompatibility and tissue specificity within the IVD microenvironment, giving them unique advantages in restoring disc homeostasis. However, current research on NP‐Exo remains limited, and their precise mechanisms of action and delivery strategies require further elucidation. Utilizing these exosomes as therapeutic carriers offers a novel approach for precisely modulating the degenerative disc microenvironment. Notably, when combined with other treatment modalities, NP‐Exo may act synergistically to more effectively target the multifactorial pathogenic processes of IVDD. However, to achieve sustained release of the therapeutic agents, we have also developed an innovative anti‐swelling hydrogel as a carrier for drug and exosome delivery. Unlike traditional hydrogels that absorb water and swell, potentially compressing disc tissue and exacerbating disc herniation, our anti‐swelling hydrogel mitigates this risk while ensuring the controlled release of therapeutic agents. This novel approach not only improves the delivery system for anti‐aging drugs and exosomes but also addresses a critical challenge in the treatment of IVDD.

**Scheme 1:**
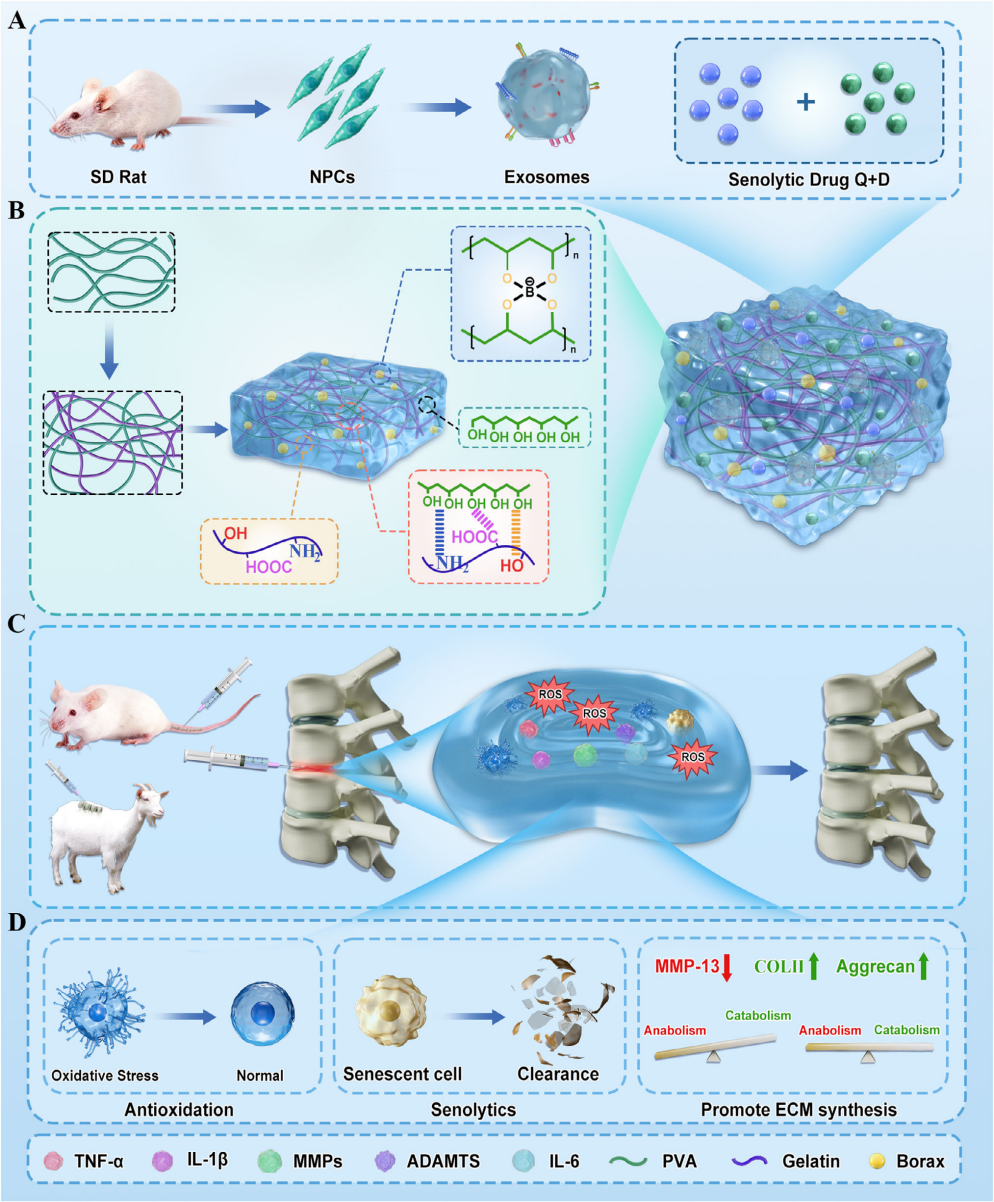
Schematic illustration of drug‐loaded hydrogel system for treatment of IVDD. A) Drug components loaded in the hydrogel. B) Constituent chemical structures and interactions of the drug‐loaded hydrogel system. C) The in‐situ treatment of the drug‐loaded hydrogel system in the rat and goat IVDD model. D) The potential mechanism of the drug‐loaded hydrogel system in the rat IVDD model.

The combined use of anti‐aging drugs, NP‐Exo and the anti‐swelling hydrogel offers a promising strategy to target the vicious cycle of senescence, oxidative stress, and inflammation, while promoting NPCs regeneration. This approach could restore NPCs function, slow disc degeneration, and ultimately offer a new avenue for the treatment of IVDD and LBP. In conclusion, targeting the mechanisms underlying NPCs degeneration through anti‐aging strategies, exosome‐based therapies, and advanced hydrogel delivery systems presents a novel and promising approach to repair IVDD and alleviate the burden of LBP (**Scheme** **1**).

## 2 Results and Discussion

### 2.1 Synthesis and Characterizations of Anti‐Swelling Hydrogel and Characterization of Exosomes

**Figure 1:**
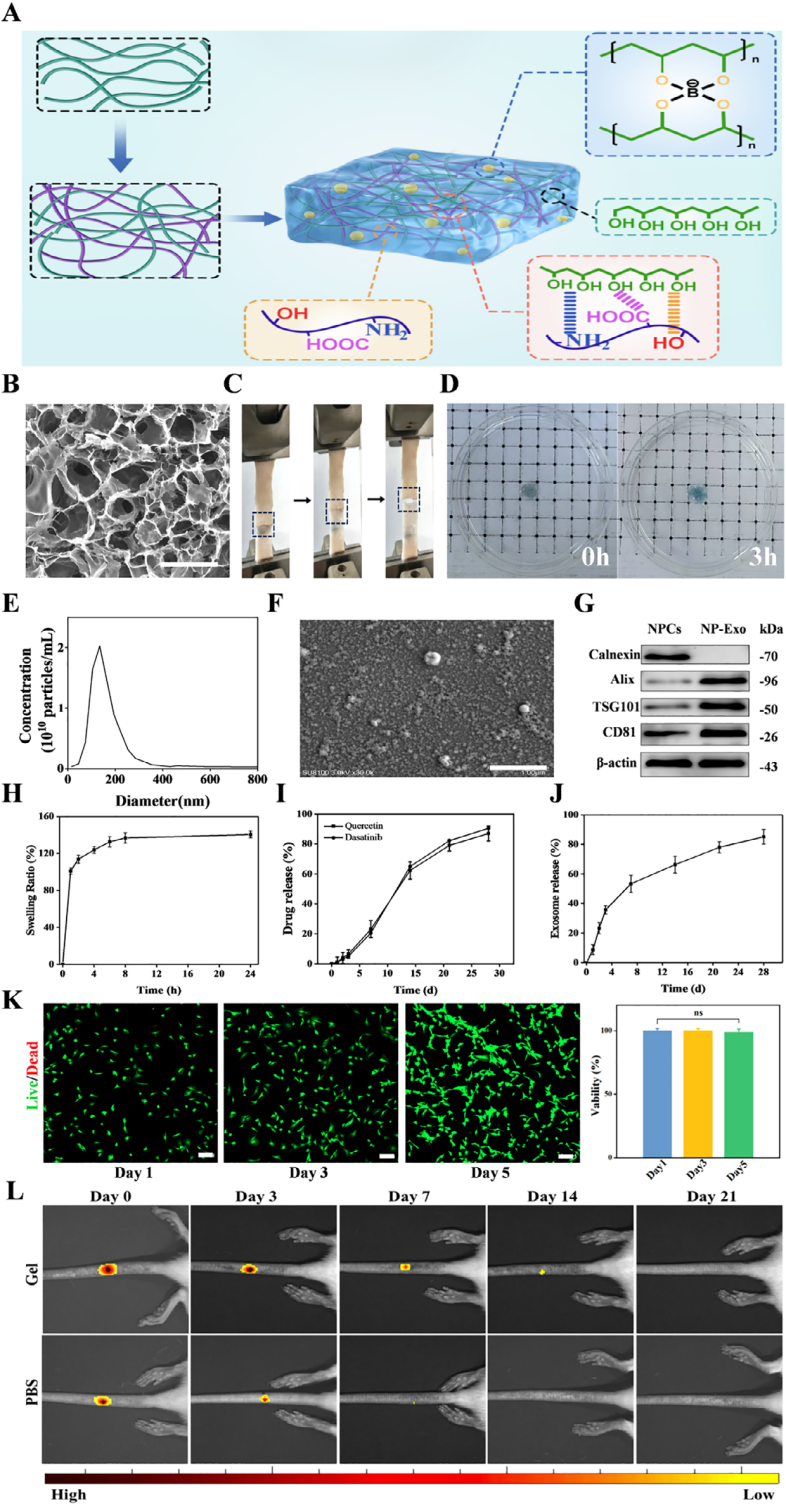
Preparation and identification of anti‐swelling hydrogel and characterization of exosomes. A) Schematic illustration of the synthesis process of anti‐swelling hydrogel. B) Typical SEM images of anti‐swelling hydrogel (Scale bar =  50 µm). C) The adhesive performance of anti‐swelling hydrogels. D) Characterization of anti‐swelling performance. E) NTA analysis of exosomes. F) SEM analysis of exosomes (Scale bar =  100 µm). G) Exosome characterization of Alix, TSG101, CD81 and Calnexin by WB. H) Water absorption swelling curve. I) Release curves of two drugs in the drug delivery system. J) Release curves of exosome in the drug delivery system. K) Biocompatibility of hydrogels was evaluated by live‐dead cell staining (Scale bar =  50 µm). L) IVIS images of the distribution of Cy5‐labeled hydrogel within the IVD.

IVDD remains a significant clinical challenge, with limited therapeutic options capable of halting its progression or facilitating tissue regeneration.^[^ ^21^ ^]^ The pathological mechanisms underlying IVDD are highly complex, involving oxidative stress, chronic inflammation, cellular senescence, and ECM degradation.^[^ ^22^ ^]^ These interrelated processes create a vicious cycle that accelerates disc degeneration, ultimately leading to structural and functional impairment of the IVD.^[^ ^23^, ^24^ ^]^ Given these multifactorial pathological drivers, there is an urgent need for innovative therapeutic strategies that can simultaneously target multiple mechanisms. In this study, we developed a novel anti‐swelling hydrogel system encapsulating senolytic agents (D+Q) and NP‐Exo to address these pathological processes in a comprehensive manner. Our findings demonstrate that this multifunctional strategy effectively mitigates oxidative stress, attenuates inflammation, inhibits cellular senescence, and promotes ECM synthesis, offering a promising therapeutic avenue for IVDD. As shown in **Figure** **1**A, a hydrogel with anti‐swelling performance was successfully constructed. Hydrogels, as hydrophilic polymers, are widely applied across various fields. In biomedical engineering, they hold great potential in areas such as soft robotics, artificial organs, and regenerative medicine.^[^ ^25^ ^]^ In the field of IVDD, hydrogels are widely used for drug delivery, cell carriers, and tissue scaffolds. With excellent biocompatibility and tunable physicochemical properties, hydrogels can create a localized microenvironment within the degenerated disc, enable sustained drug release, and support cell adhesion and survival, thereby promoting tissue repair and functional restoration. These advantages highlight their broad application prospects in IVDD therapy.^[^ ^26^, ^27^, ^28^ ^]^ The SEM images show that the surface of the hydrogel presents a uniformly distributed porous structure (Figure 1B), which is conducive to the loading and sustained release of the drug. An ideal anti‐adhesion hydrogel should achieve durable and stable adhesion on the surface of degenerated intervertebral disc tissue. To quantitatively evaluate its adhesive properties, this study utilized porcine skin tissue to simulate the in vivo environment and conducted systematic measurements using a lap shear test combined with a biomechanical testing system. The results demonstrated that the hydrogel achieved an adhesive strength of 4.18 kPa (Figure 1C), confirming its excellent interfacial adhesion capability, which enables firm attachment to the target tissue area while effectively preventing drug leakage. Despite their extensive exploration for drug delivery in IVDD treatment, conventional hydrogels are constrained by the limited anatomical space of the IVD. Their inherent water‐absorbing and swelling characteristics often lead to localized pressure elevation, potentially exacerbating disc degeneration. To overcome these limitations, our hydrogel incorporates a dual‐network structure that not only minimizes swelling but also enables the controlled release of therapeutic agents. The evaluation results of the anti‐swelling performance showed that the swelling rate of this hydrogel after soaking in PBS for 24 h was only 1.4 (Figure 1D,H), which was significantly better than that of the traditional hydrogel and overcame the defect of the latter, which compressed the surrounding tissues and aggravated IVD protrusion due to water absorption and swelling. In terms of exosome characterization, the results of NTA analysis showed that the particle size of the extracted exosomes presented a unidirectional distribution, mainly ranging from 30 to 200 nm, which was consistent with previous reports (Figure 1E). SEM observation showed that exosomes derived from NPCs presented typical cup‐shaped or circular structures (Figure 1F). WB detection indicated that exosomes expressed the typical marker proteins Alix, TSG101 and CD81, but did not express the negative marker protein Calnexin, further verifying the accuracy and purity of their source (Figure 1G). The release performance evaluation shows that the hydrogel system can continuously release most of the loaded drugs and exosomes within 28 days, demonstrating excellent sustained‐release ability (Figure 1I,J). The results of the cytocompatibility experiment showed that NPCs maintained good viability after being cultured in the hydrogel for 1, 3, and 5 days, with the cell number increasing over the incubation time. Moreover, the NPCs exhibited high cell viability (>90%), indicating that this material has excellent biocompatibility. (Figure 1K). In addition, to evaluate in vivo biocompatibility of the hydrogel, major organs were harvested 8 weeks after intradiscal injection and subjected to H&E staining (Figure S2, Supporting Information). To evaluate the in vivo degradation profile of the hydrogel, Cy5‐labeled G‐CS hydrogel was injected into rat IVDs and monitored using an in vivo imaging system, with Cy5‐labeled PBS injections serving as the control group (Figure 1L).

The results show that the hydrogel can stably remain in the body for at least 21 days, demonstrating the characteristics of slow degradation and continuous drug release, which is helpful for prolonging the therapeutic time of the drug in the target area.

### 2.2 Anti‐Apoptotic and Anti‐Senescence Properties

**Figure 2:**
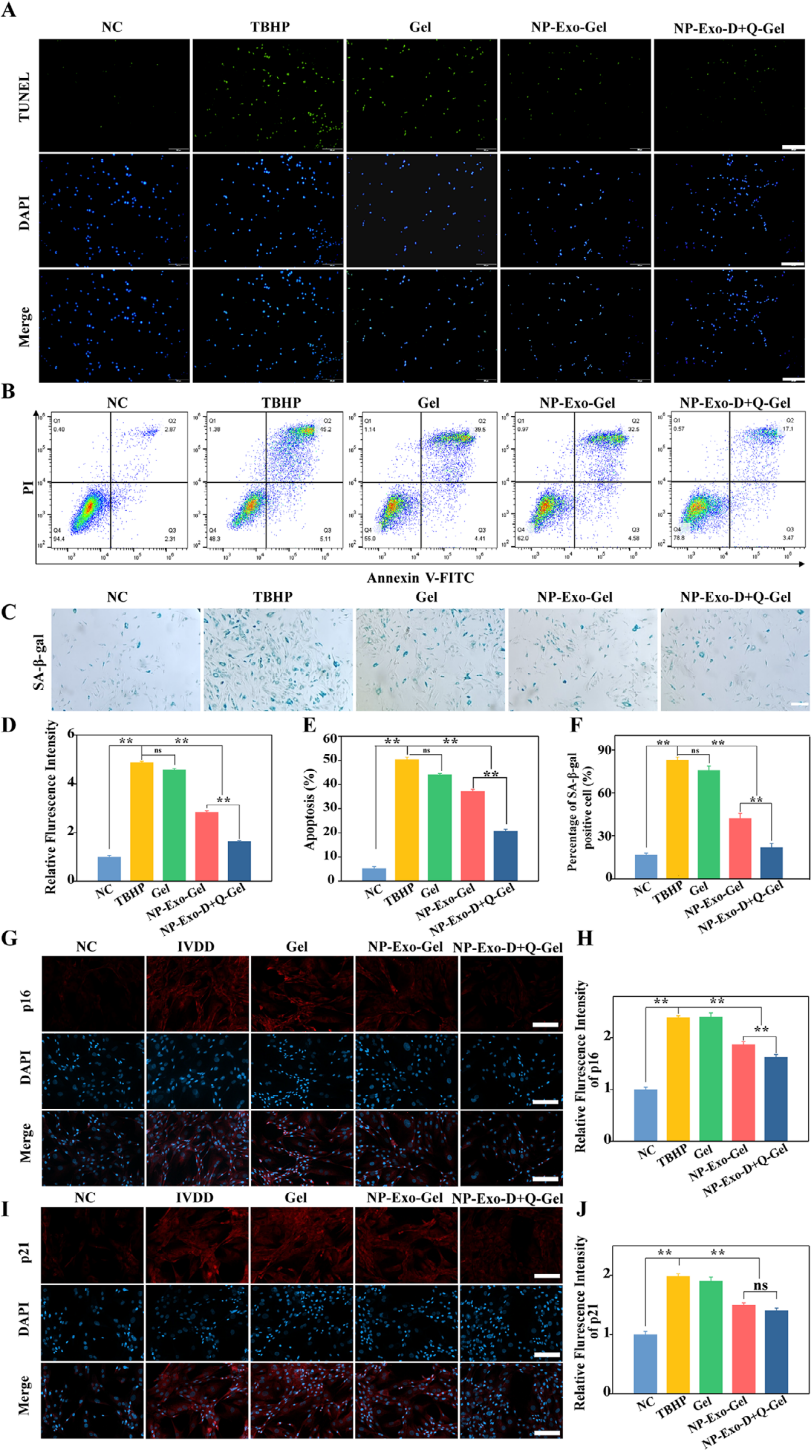
Anti‐apoptotic and anti‐senescence properties. A) Representative images of TUNEL staining in TBHP‐treated cells incubated with different materials (n = 3, scale bar: 50 µm). B) Flow cytometry analysis of TBHP‐treated cells incubated with different materials. C) Representative images of SA‐β‐gal staining in TBHP‐treated cells incubated with different materials (n = 3, scale bar: 50 µm). D) Semi‐quantitative analysis of fluorescence intensity of TUNEL (n = 3). E) Quantification of flow cytometry results. F) Quantitative analysis of the percentage of SA‐β‐gal positive cells (n = 3). G,H) Immunofluorescence staining of p16 and relative fluorescence intensity analysis in TBHP‐treated cells incubated with different materials (n = 3, scale bar: 50 µm). I,J) Immunofluorescence staining of p21 and relative fluorescence intensity analysis in TBHP‐treated cells incubated with different materials (n = 3, scale bar: 50 µm). **p< 0.01.

IVDD is closely associated with the apoptosis and senescence of NPCs.^[^ ^29^, ^30^ ^]^ Notably, senescent cells often exhibit a senescence‐associated secretory phenotype (SASP), releasing various pro‐inflammatory cytokines and matrix‐degrading enzymes that exacerbate local inflammation and ECM degradation.^[^ ^31^ ^]^ Given the critical role of NPCs in maintaining disc homeostasis, preventing their senescence and apoptosis is paramount.^[^ ^32^ ^]^ Studies have shown that the classic combination of D+Q can effectively clear senescent cells and extend lifespan in mice, making it a well‐recognized senolytic therapy.^[^ ^33^ ^]^ In the field of IVDD, however, the application of D+Q has so far been limited to oral administration, with no reports of direct injection into the IVD.^[^ ^19^ ^]^ First, a series of preliminary experiments were performed to identify the optimal concentrations and ratio of quercetin and dasatinib for combination therapy. The findings demonstrated that the combination of quercetin (2 µM) and dasatinib (10 nM) yielded the most pronounced effects in terms of anti‐aging properties and ECM regulation. Consequently, this specific combination was chosen as the treatment condition for subsequent experiments (Figure S1, Supporting Information). We next employed a combination of TUNEL staining, flow cytometry, and SA‐β‐gal staining to evaluate apoptosis and senescence in NPCs (**Figure** **2**A–C). Our experimental results revealed that, compared with the control group, TBHP treatment markedly enhanced apoptosis in NPCs, as indicated by a significant increase in the proportion of TUNEL‐positive cells (*p* < 0.01) and a substantial rise in the percentage of senescent cells, suggesting that TBHP induction aggravated cellular aging. Flow cytometry analysis further corroborated a pronounced elevation in the proportion of apoptotic cells following TBHP exposure. Treatment with NP‐Exo significantly mitigated both the TUNEL‐positive rate and the apoptotic population detected by flow cytometry, as well as the proportion of SA‐β‐gal‐positive senescent cells, thereby exhibiting robust anti‐apoptotic and anti‐senescence effects. Notably, in the group receiving combined treatment with D+Q the proportions of positive cells in both TUNEL and SA‐β‐gal staining were further reduced (*p* < 0.01), and the lowest apoptosis rate was observed in flow cytometry analysis, indicating that combination therapy more effectively suppressed apoptosis and senescence, thus enhancing therapeutic efficacy (Figure 2D–F). Furthermore, immunofluorescence staining demonstrated that TBHP markedly upregulated the expression of the senescence‐associated genes p16 and p21, whereas exosome treatment significantly downregulated their expression (*p* < 0.01); notably, in the group treated with a combination of D+Q, the expression levels of p16 were restored even more significantly (*p* < 0.01) (Figure 2G–J). These findings indicate that the combined application of exosomes with D+Q effectively inhibits TBHP‐induced apoptosis and senescence of NPCs, thereby providing a novel therapeutic strategy for IVDD.

### 2.3 ECM Expression Analysis

**Figure 3:**
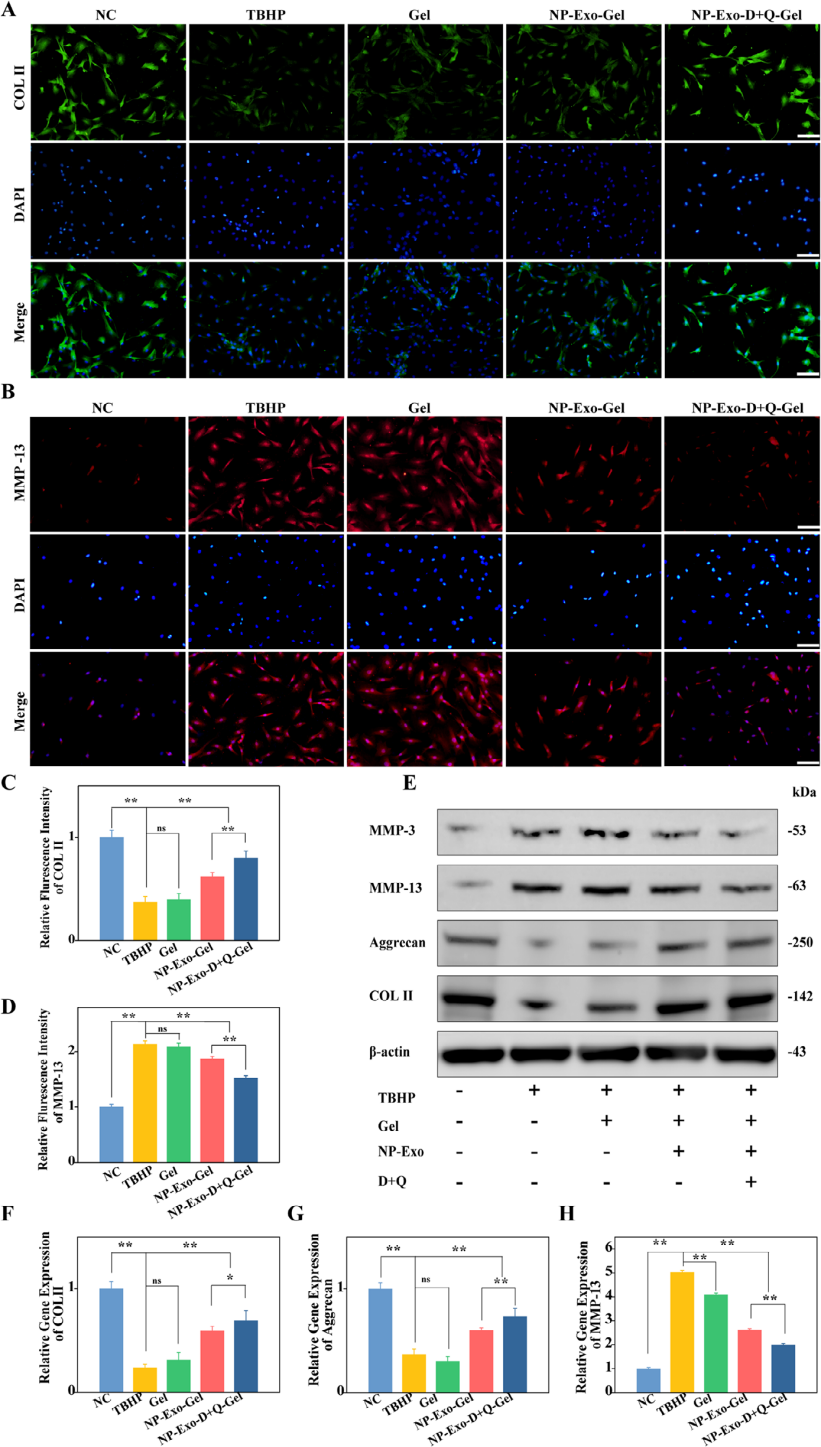
Regulatory role of the ECM. A) Immunofluorescence staining of COL II in TBHP‐treated cells incubated with different materials (n = 3, scale bar: 100 µm). B) Immunofluorescence staining of MMP‐13 in TBHP‐treated cells incubated with different materials (n = 3, scale bar: 100 µm). C) Semi‐quantitative analysis of fluorescence intensity of COL II (n = 3). D) Semi‐quantitative analysis of fluorescence intensity of MMP‐13 (n = 3). E) WB analysis of ECM synthesis‐related proteins in TBHP‐treated cells incubated with different materials. F–H) Relative expression levels of COL II, Aggrecan, and MMP‐13 in TBHP‐treated cells cultured with different materials. Data are expressed as the mean ± SD (n = 3). *p< 0.05; **p< 0.01.

Because ECM degradation is a key pathological feature of IVDD, restoring ECM homeostasis is crucial for successful therapeutic intervention.^[^ ^4^ ^]^ Our data provide compelling evidence that NP‐Exo, in combination with D+Q, not only enhances ECM synthesis but also inhibits its degradation. To evaluate the effects of NP‐Exo and the combination of D+Q on ECM synthesis, we conducted immunofluorescence staining to assess the expression of COL II, an ECM synthesis‐related protein, and MMP‐13, an ECM degradation‐related protein. Additionally, WB analyses and qPCR were performed to quantify the expression levels of Aggrecan, COL II, MMP‐13, and MMP‐3. The immunofluorescence images (**Figure** **3**A,B) and the semi‐quantitative analysis of fluorescence intensity indicated that (Figure 3C,D), relative to the control group, the TBHP‐treated group exhibited a significant reduction in COL II expression and a marked increase in MMP‐13 expression. Conversely, the NP‐Exo intervention group demonstrated not only a partial restoration of COL II expression and reconstruction of the extracellular mesh‐like structure, but also a decrease in MMP‐13 expression levels. Notably, the group treated with the combination of D+Q showed a further significant increase in COL II expression exhibiting a characteristic extracellular mesh‐like distribution pattern, along with a more pronounced reduction in MMP‐13 expression (*p* < 0.01). These findings were corroborated by WB analyses, which yielded consistent results (Figure 3E). Furthermore, to support these observations at the transcriptional level, we performed real‐time PCR analysis of ECM‐related genes, including COL II, Aggrecan, and MMP‐13, which revealed expression trends consistent with the protein‐level findings (Figure 3F–H). Collectively, these data suggest that NP‐Exo, in conjunction with D + Q, effectively promote ECM synthesis and inhibit its degradation, thereby exerting a synergistic protective effect on the ECM. These molecular changes suggest that the combination therapy not only restores ECM balance at the cellular level but also improves the biomechanical properties of the disc, which is essential for long‐term functional restoration.

### 2.4 Antioxidation and Anti‐Inflammation Evaluation

The above experiments have confirmed that the combined application of D+Q with NP‐Exo effectively reduces apoptosis and senescence of NPCs and promotes ECM synthesis in the IVD. To preliminarily explore the mechanisms underlying these biological effects, we subsequently conducted further experiments. Oxidative stress and inflammation are two critical driving factors in IVDD,^[^ ^34^ ^]^ forming a vicious cycle that accelerates disc degeneration.^[^ ^35^, ^36^ ^]^ Excessive accumulation of ROS damages cellular DNA, proteins, and lipids, triggering apoptosis and senescence, which in turn accelerate disc degeneration.^[^ ^37^ ^]^ Concurrently, inflammatory responses mediated by injury‐induced pro‐inflammatory cytokines (e.g., IL‐1β, TNF‐α, IL‐6) further promote ECM degradation, apoptosis, and fibrosis. These processes interact in a vicious cycle, exacerbating disc tissue degeneration.

**Figure 4:**
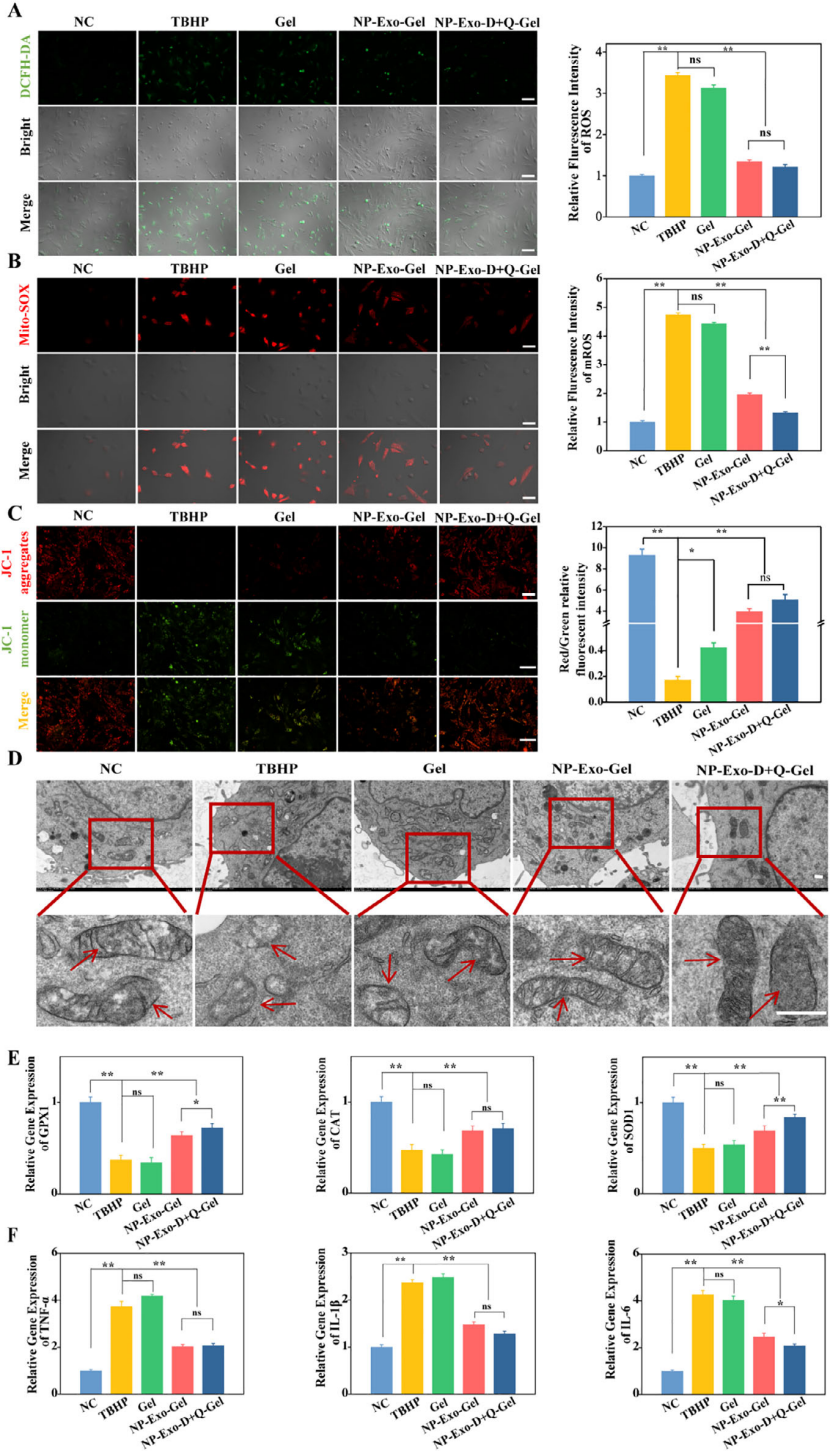
Assessment of Antioxidant and Anti‐inflammatory Activities. A) Representative images of NPCs detected by DCFH‐DA fluorescence staining (n = 3, scale bar: 100 µm). B) Mito‐SOX staining of NPCs under different treatment conditions (n = 3, scale bar: 100 µm). C) Representative fluorescent images of JC‐1 staining under different treatment conditions (n = 3, scale bar: 100 µm). D) TEM images of mitochondria in NPCs under different treatment conditions (n = 3, scale bar: 500 nm). E) Relative expression levels of GPX1, SOD1, and CAT in TBHP‐treated cells cultured with different materials. F) Relative expression levels of IL‐1β, TNF‐α, and IL‐6 in TBHP‐treated cells cultured with different materials. Data are expressed as the mean ± SD (n = 5). *p< 0.05; **p< 0.01.

To evaluate the anti‐inflammatory and antioxidant effects of NP‐Exo in combination with quercetin and dasatinib, we first assessed the clearance of intracellular ROS using the DCFH‐DA staining kit. TBHP‐treated NPCs exhibited pronounced green fluorescence, confirming the successful establishment of an oxidative stress model. The introduction of NP‐Exo reduced the fluorescent signal, indicating attenuation of TBHP‐induced oxidative stress, while the further addition of quercetin and dasatinib led to a significant reduction in fluorescence, suggesting an enhanced antioxidant effect with the combined treatment (**Figure** **4**A).

Given that oxidative stress is usually closely related to mitochondrial function, and mitochondria are both the main source of ROS generation and the targets of their attacks, we further evaluated the oxidative stress and functional status at the mitochondrial level. First, Mito‐SOX staining was used to detect mitochondrial reactive oxygen species (mtROS). The results showed that the combined treatment group significantly reduced the accumulation of mtROS (Figure 4B). Subsequently, we analyzed the changes in mitochondrial membrane potential (MMP) using the JC‐1 staining method. The results showed that after TBHP treatment, the proportion of JC‐1 monomers (green) in the cells increased and the aggregate (red) decreased, indicating a decrease in MMP. However, NP‐Exo combined with quercetin and dasatinib treatment could significantly restore the red/green fluorescence ratio. It is indicated that the MMP has been effectively maintained (Figure 4C). Subsequently, the ultrastructure of mitochondria was observed through TEM. It was found that the combined treatment significantly improved the morphological characteristics of mitochondria. The membrane structure was intact and the cristae were clear, suggesting that the mitochondrial function was effectively protected (Figure 4D).

Moreover, qPCR analysis of the antioxidant genes CAT, GPX1, and SOD1 revealed that TBHP treatment significantly downregulated their expression. Intervention with NP‐Exo partially restored gene expression, and the combined treatment with D+Q further enhanced this recovery, demonstrating a synergistic effect in bolstering cellular antioxidant defenses (Figure 4E). To validate the anti‐inflammatory efficacy, we measured the mRNA levels of IL‐1β, TNF‐α, and IL‐6. TBHP markedly upregulated these cytokines, whereas NP‐Exo treatment reduced their expression, with the combined treatment further suppressing the inflammatory response (Figure 4F). In summary, our findings reveal that the combination of quercetin and dasatinib with NP‐Exo synergistically enhances both antioxidant and anti‐inflammatory capabilities. This approach not only effectively decreases intracellular ROS levels and alleviates oxidative damage but also markedly suppresses the expression of pro‐inflammatory cytokines, thereby mitigating inflammatory microenvironment implicated in disc degeneration. Moreover, the combined treatment significantly improves mitochondrial morphology, preserves mitochondrial membrane potential and structural integrity, indicating its substantial role in safeguarding mitochondrial function. These findings highlight that our therapeutic strategy effectively disrupts the vicious cycle of oxidative stress and inflammation by protecting mitochondrial function, thereby slowing IVDD progression and preserving disc function.

### 2.5 X‐Ray and MRI‐Based Imaging Assessment of The Rat IVDD Model

**Figure 5:**
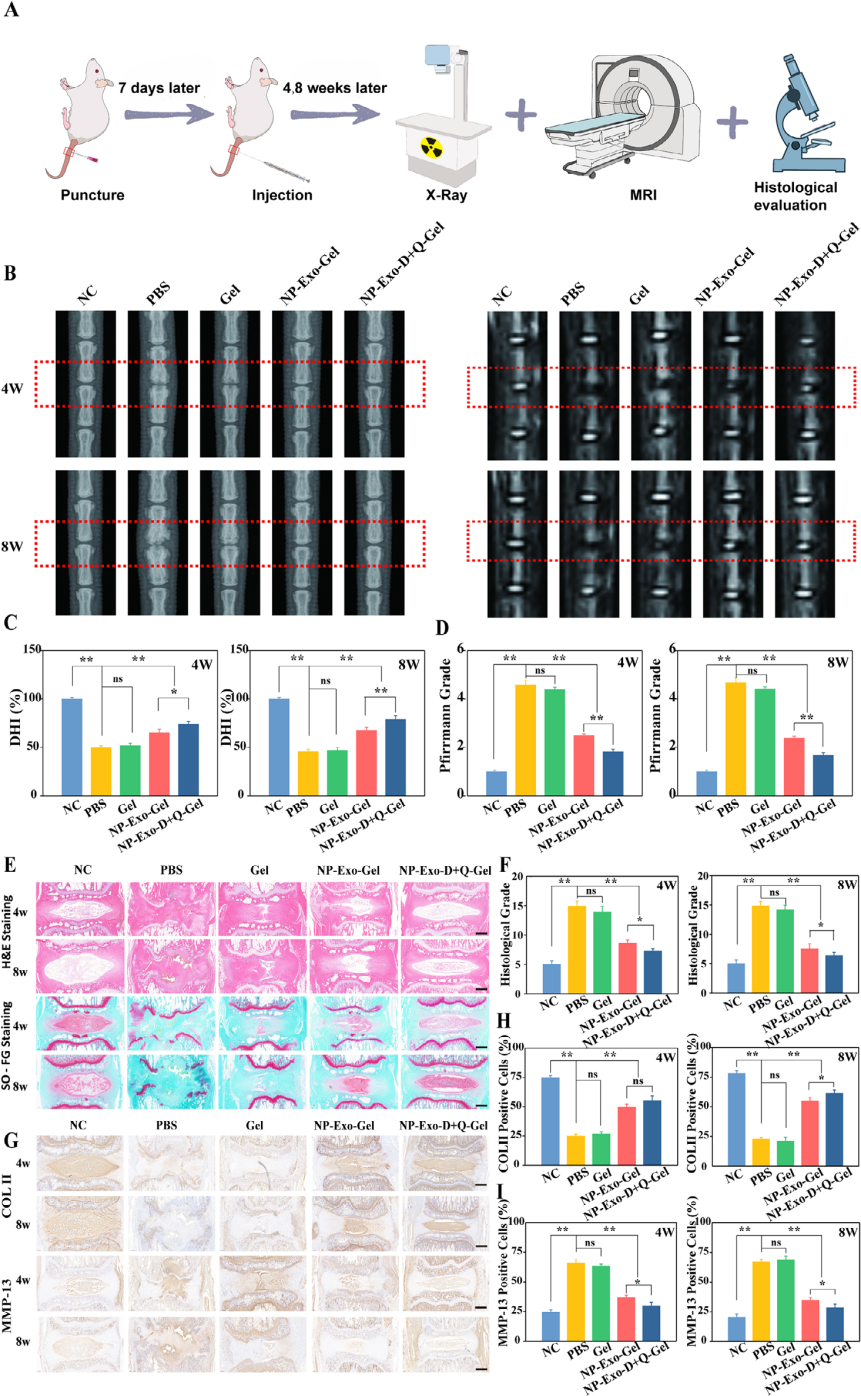
Radiological and histological evaluations in a needle puncture‐induced rat IVDD model demonstrated representative imaging and tissue changes. A) Summary of animal experiments in the rat model. B) Representative X‐ray and MRI images of IVD in the five groups at 4 and 8 weeks post‐treatment. C,D) Statistical analyses of DHI% and MRI grading among different groups at 4 and 8 weeks post‐treatment. E) The H&E and SO‐FG staining images of different groups at 4 and 8 weeks post‐treatment (n = 3, scale bar: 1 mm). F) Histological grading of different groups at 4 and 8 weeks post‐treatment. G) COL II and MMP‐13 expression in the rat IVDD model at 4 and 8 weeks post‐treatment (n = 3, scale bar: 1 mm). H) The percentage of COL II‐positive cells in immunohistochemical staining at 4 and 8 weeks post‐surgery. I) The percentage of MMP‐13‐positive cells in immunohistochemical staining at 4 and 8 weeks post‐surgery. Data are expressed as the mean ± SD (n = 5). *p < 0.05; **p < 0.01.

To evaluate the potential therapeutic effects of NP‐Exo combined with Q+D on IVDD, we established a rat IVDD model via needle puncture (**Figure** **5**A). Radiological assessments of the rat IVD tissues were conducted at 4 and 8 weeks post‐treatment. Specifically, the degree of disc degeneration was assessed by calculating the disc height index percentage (DHI%) from X‐ray images, and histological and signal changes in the IVD were evaluated using MRI (Figure 5B).

At 4 weeks post‐treatment, the puncture‐treated group exhibited a significant reduction in DHI% (Figure 5C), with MRI scans revealing decreased signal intensity, indicating a higher degree of disc degeneration (Figure 5D). The formula for calculating DHI% is shown in (Figure S3, Supporting Information). In contrast, the group receiving NP‐Exo intervention showed partial restoration of DHI% and increased MRI signal intensity, suggesting improvements in disc structure and function. Notably, the D+Q combination therapy group demonstrated a more pronounced recovery in DHI% (Figure 5C), with MRI signals approaching normal levels, indicating effective suppression of disc degeneration (Figure 5D).

By the 8‐week evaluation, disc degeneration in the puncture‐treated group had further progressed, with a continued decline in DHI% (Figure 5C) and further diminished MRI signals (Figure 5D). Conversely, both the exosome intervention group and the D+Q combination therapy group exhibited further improvements in DHI% and corresponding increases in MRI signal intensity. Notably, the D+Q combination therapy group nearly restored DHI% and MRI signals to normal levels, demonstrating the most significant therapeutic effect. These findings suggest that NP‐Exo combined with D+Q exert a synergistic effect in repairing IVDD, with therapeutic efficacy evident at 4 weeks and further consolidated and enhanced by 8 weeks.

### 2.6 Histological Analysis of The Rat IVDD Model

To further validate that NP‐Exo combined with D+Q can repair IVDD by inhibiting local inflammatory responses in the degenerative microenvironment and reducing NPCs senescence, we conducted histological analyses. H&E staining results (Figure 5E) showed that at week 4, the NP‐Exo group exhibited clearer disc tissue structures compared to the PBS group, with uniform internal structures of the nucleus pulposus and relatively distinct annulus fibrosus boundaries. The NP‐Exo combined with D+Q group demonstrated even more pronounced improvements. By week 8, the PBS group displayed more severe degeneration, whereas the NP‐Exo combined with D+Q group showed significant repair effects. Safranin O and Fast Green staining (Figure 5E)  indicated that at both 4 and 8 weeks, the NP‐Exo group mitigated the loss of proteoglycan‐rich matrix in the nucleus pulposus caused by puncture, with the NP‐Exo combined with D+Q group exhibiting the least matrix loss. Histological scoring (Figure 5F) revealed that the NP‐Exo combined with D+Q group had a more significant therapeutic effect than the NP‐Exo group, effectively delaying puncture‐induced disc degeneration, with statistically significant differences compared to the PBS group (*p* < 0.01).

Immunohistochemical analysis showed that compared to the PBS group, the NP‐Exo combined with D+Q group had significantly increased COL II signal intensity at 4 and 8 weeks, and significantly downregulated MMP‐13 expression (Figure 5G). Positive cell rate analysis further supported these therapeutic effects (Figure 5H,I). These results indicate that NP‐Exo combined with D+Q can effectively repair IVDD by inhibiting local inflammatory responses and reducing NPCs senescence. Moreover, the therapeutic efficacy of NP‐Exo combined with D+Q is superior to that of NP‐Exo alone.

### 2.7 X‐Ray, MRI‐Based Imaging Assessment and Histological Analysis of The Goat IVDD Model

**Figure 6:**
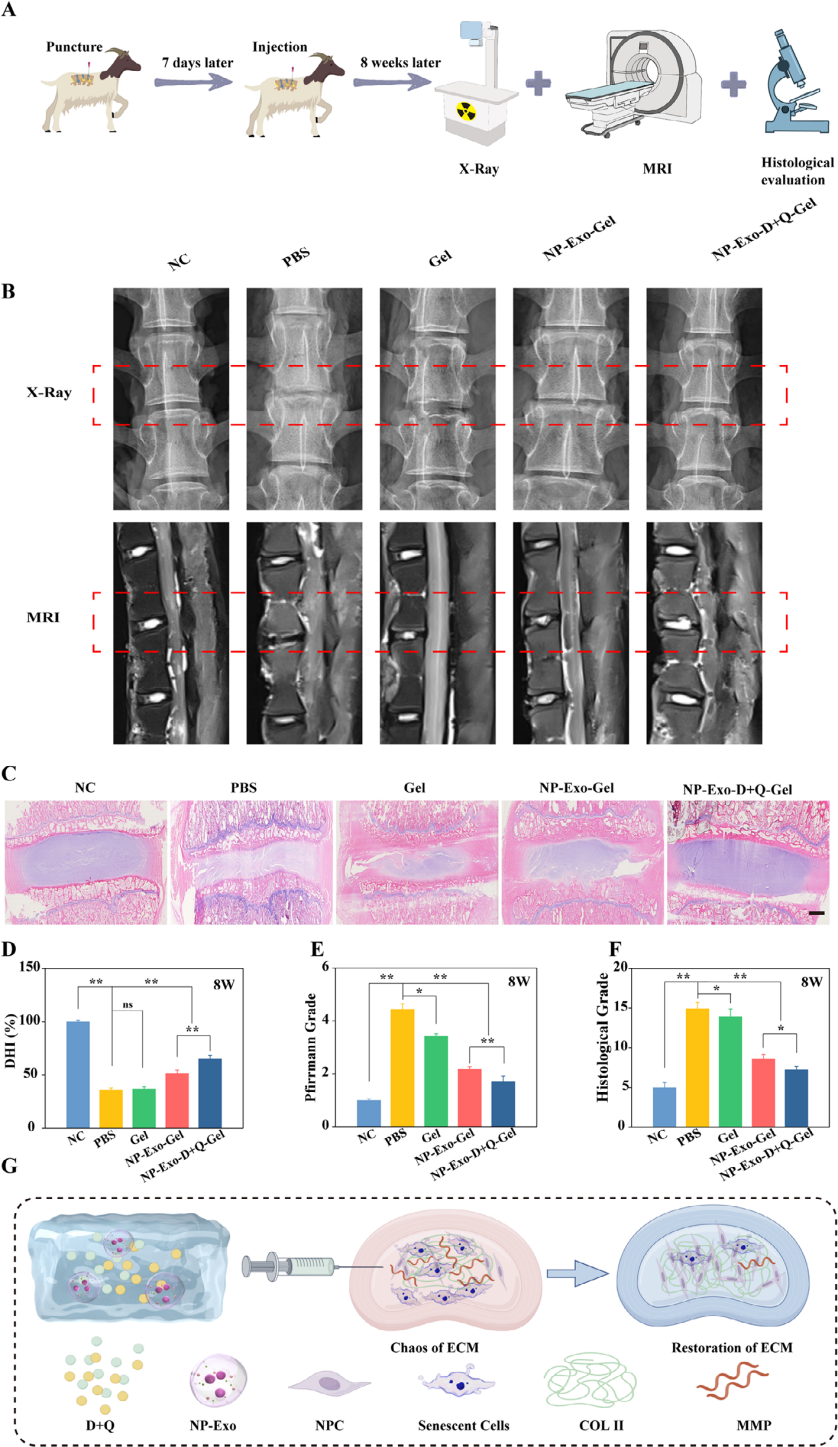
Radiological imaging and histological assessment in a needle puncture‐induced goat IVDD model. A) Summary of animal experiments in the goat model. B) Representative X‐ray and MRI images of IVD in the five groups at 8 weeks post‐treatment. C) The H&E staining images of different groups at 8 weeks post‐treatment. D) Statistical analysis of the DHI% between different groups at 8 weeks post‐treatment. E) Statistical analysis of the MRI grading between different groups at 8 weeks post‐treatment. F) Histological grading of different groups at 8 weeks post‐treatment. G) Schematic illustration of the hydrogel restoring the ECM. Data are expressed as the mean ± SD (n = 5). *p< 0.05; **p< 0.01.

Given the translational significance of our findings, we specifically extended the study to a large animal model with higher clinical relevance‐the goat IVDD model. The anatomical structure, physiological loading, and degeneration process of goat IVDs are more similar to those of humans, making this model highly representative and valuable for preclinical research in IVDD.^[^ ^38^ ^]^ Therefore, we employed a puncture‐induced goat IVDD model (**Figure** **6**A). 8 weeks post‐treatment, we conducted imaging assessments of the goat IVD tissues, utilizing the same evaluation parameters as in the rat studies (Figure 6B). As shown in Figure 6D,E, the results indicated that the puncture‐treated group exhibited a significant decrease in the DHI% on X‐ray images, suggesting a notable reduction in disc height. MRI evaluations revealed a marked decrease in signal intensity in the TBHP group, indicating exacerbated disc degeneration. In contrast, goats receiving NP‐Exo interventions demonstrated improvements in both DHI% and MRI signal intensity, suggesting a degree of inhibition in disc degeneration. Notably, the group treated with the combination of D+Q exhibited even higher DHI% and stronger MRI signals, indicating more pronounced restoration of disc structure and function.

In addition to imaging analysis, we also performed H&E staining on goat disc tissues to evaluate histological changes. The results showed that the NP‐Exo group displayed better preservation of disc structure with more organized nucleus pulposus and annulus fibrosus compared to the TBHP group. Remarkably, the D+Q combination therapy group demonstrated the most intact disc architecture and minimal histological signs of degeneration (Figure 6C,F). These findings provide compelling evidence supporting the efficacy of NP‐Exo combined with D+Q in promoting IVD repair in a goat model, thereby reinforcing their potential therapeutic application in IVDD.

In summary, beyond its therapeutic efficacy, the minimally invasive nature of our hydrogel‐based delivery system presents a key advantage over conventional IVDD treatment strategies. By offering a biocompatible and effective platform for the localized and sustained release of senolytic drugs and NP‐Exo, this approach directly targets the root causes of disc degeneration while minimizing systemic side effects. Consequently, this strategy holds substantial promise for integration into clinical practice as a viable alternative to existing treatment modalities for IVDD and LBP. Despite the promising therapeutic potential demonstrated in our study, we acknowledge certain limitations, particularly regarding the long‐term efficacy of the hydrogel system. Given that IVDD is a chronic degenerative condition requiring sustained therapeutic effects, future investigations should focus on assessing the long‐term stability, safety, and efficacy of this system. Additionally, further optimization of hydrogel formulations, exploration of additional therapeutic agents, and large‐scale preclinical and clinical studies will be essential for advancing this technology toward clinical translation in the management of chronic IVDD.

## 3 Conclusion

In conclusion, our study demonstrates that the combination of NP‐Exo, D+Q delivered via an anti‐swelling hydrogel system, effectively targets the key pathological mechanisms of IVDD, including cellular senescence, inflammation, oxidative stress, and ECM degradation. This multifunctional approach not only restores NPCs function and ECM homeostasis but also provides a promising therapeutic strategy for the treatment of IVDD and LBP. By addressing the root causes of disc degeneration, this novel therapy has the potential to significantly improve patient outcomes and reduce the socioeconomic burden associated with LBP.

## 4 Experimental Section

### Preparation and Characterizations of Anti‐Swelling Hydrogel

PVA (190 mg) was dissolved in 2 mL DI water by heating, then added 32 mg gelatin and 2 mg borax, subsequently, 200 µL genipin solution (1%) was added above composite solution to prepared hydrogel. The adhesive strength of the hydrogel was evaluated using an in vitro shear bonding test on porcine skin. Pre‐treated skin samples were bonded to the hydrogel with a fixed contact area under preload pressure. A universal testing machine applied shear force at a constant displacement rate until failure occurred. The maximum shear stress was recorded, and the failure mode was characterized. The morphology of hydrogel was tested through scanning electron microscope (SEM).

### Isolation of Rat NPCs

NPCs were isolated from 6–8 week‐old Sprague‐Dawley rats euthanized by cervical dislocation. Under sterile conditions, caudal vertebrae were dissected and IVDs were carefully extracted. After removing the annulus fibrosus, nucleus pulposus tissue was separated using sterile surgical instruments and digested with 0.2% collagenase II at 37 °C for 2–4 h. The digested tissue was filtered through a 70 µm cell strainer, centrifuged at 1500 rpm for 10 min, and resuspended in complete DMEM/F12 medium supplemented with 10% fetal bovine serum. Primary NPCs were cultured at 37 °C with 5% CO_2_, with medium changes every 2–3 days. Cells at passage 3 were used for subsequent experiments.

### Isolation and Identification of NP‐Exo

Exosome isolation from NPCs by differential ultracentrifugation. Briefly, the NPCs were seeded at 1 × 10^6^ cells per T75 flask and cultured until 80% confluence. The medium was replaced with exosome‐depleted complete medium, and after 48 h, the conditioned medium was collected. Differential ultracentrifugation was performed at 4 °C, starting with 300 g for 10 min to remove floating cells, followed by 2000 g for 10 min to eliminate residual cells, and 10 000 g for 30 min to remove debris. Exosomes were then isolated by ultracentrifugation at 100 000 g for 70 min. The pellet was resuspended in PBS, filtered through a 0.22 µm filter, and subjected to a second round of ultracentrifugation. The final exosome pellet was resuspended in 100 µL PBS and stored at −80 °C. Exosome particle size was analyzed using nanoparticle tracking analysis (NTA) with a ZetaVIEW S/N 17–310 (PARTICLE METRIX). The morphology of NP‐Exo was further examined by transmission electron microscopy (TEM). Exosome characterization was confirmed by Western blot (WB) analysis for the expression of specific exosomal markers. Specifically, WB was used to identify exosomes by detecting specific marker proteins (CD81, Alix, Calnexin, TSG101).

### Profile of Exosome Release from The Hydrogels

The release of exosomes was detected using the Rat CD81 ELISA Kit (Absin, Shanghai, China) provided by Absin (Shanghai) Biotechnology Co., Ltd. The specific operation was as follows: 100 µL of hydrogel containing 1 µg of exosomes or 100 µL of hydrogel without exosomes was placed in the upper chamber of a 24‐well transwell plate (Corning, China), and 200 µL of PBS buffer was added to the lower chamber. Then, 100 µL of PBS was sampled at 0, 1, 2, 3, 7, 14, 21, and 28 days, and immediately replenished with an equal volume of fresh PBS. The cumulative release ratio of exosomes was further calculated by detecting the amount of exosomes released at each time point.

### Screening for Optimal Concentration and Ratio of Combined Use of D and Q

The experiment to determine the optimal concentration and ratio of dasatinib (D) and quercetin (Q) for combined treatment on NPCs involves assessing the cytotoxicity of each compound using the CCK‐8 assay. NPCs were seeded into 96‐well plates at a density of 3000 cells per well and cultured for 24 h. For quercetin, concentrations of 0, 0.5, 1, 2, 4, and 8 µM were applied to the cells, and cell viability is measured after 1 and 3 days of treatment. Similarly, for dasatinib, concentrations of 0, 0.1, 1, 10, and 50 nM were used, with viability assessed after 1 and 3 days. After incubation, 10 µL of CCK‐8 reagent is added to each well, followed by 1–4 h of incubation at 37 °C. Absorbance at 450 nm was measured using a microplate reader to determine cell viability. The data are analyzed to plot concentration‐response curves for each drug and identify the optimal concentrations for each treatment. Based on the individual drug data, combination treatments of dasatinib and quercetin are tested at various ratios and concentrations to determine the best combination for further study.

### Profile of D and Q Release from The Hydrogels

Select physiological saline or PBS as the release medium and adjust the pH to 7.4 to simulate physiological conditions. Place a certain amount of hydrogel in a dialysis bag (MWCO = 1000), immerse it in the release medium, and stir it at 100 revolutions per minute on a magnetic stirrer while maintaining a constant temperature of 37 °C. At the predetermined time points, take out 3.0 mL of the release medium and add an equal volume of PBS. Analyze the concentration of the released drug by ultraviolet‐visible spectrophotometry. All release measurements were repeated three times, and the average values were plotted on a graph. Compare the amount of released drug with the initial drug amount and calculate the release rate.

### Hydrogel Degradation Experiment

A Cy5‐labeled hydrogel was administered into the IVDs of rats in suitable amounts. To investigate the degradation of hydrogel in the rat model, changes in fluorescence intensity within the IVDs were monitored on days 0, 3, 7, 10, 14, and 28 using a non‐invasive live optical imaging system for small animals (IVIS Spectrum, PerkinElmer).

### Live/Dead Cell Staining

The experiment was carried out using a Calcein‐AM/PI cell viability/cytotoxicity assay kit (Beyotime, China). Specifically, NPCs were co‐cultured with drug‐loaded hydrogels in a 12‐well plate under various stimuli for 1, 3, and 5 days. Thereafter, the cells were stained with Calcein‐AM and PI for 30 min. The stained samples were analyzed using a confocal microscope.

### Intracellular ROS Scavenging Ability Evaluation

Fand cultured for 24 h to allow cell attachment and ensure they were in the logarithmic growth phase. The cells were then treated with 50 µM TBHP for 24 h to induce oxidative stress and establish the cellular model.^[^ ^39^, ^40^ ^]^ After treatment, the medium containing TBHP was removed, and the cells were gently washed twice with PBS to remove any excess TBHP. Different experimental compounds were then added, and the cells were cultured for an additional 2 days. After treatment, the ROS levels were assessed using both DCFH‐DA and Mito‐SOX (Beyotime). For DCFH‐DA staining, the cells were incubated with the probe for 30–45 min to enable its entry into the cells and reaction with ROS. Post‐incubation, the cells were washed twice with PBS to remove unbound DCFH‐DA. For Mito‐SOX staining, the cells were similarly treated according to standard protocols. Fluorescence intensity was subsequently observed under a fluorescence microscope, and ROS levels across different treatment groups were documented. A decrease in fluorescence intensity signifies enhanced ROS scavenging capacity.

### Assessment of Mitochondrial Membrane Potential

The NPCs were plated in a 96‐well plate at a density of 8 × 10^3^ cells per well. Once the cells had fully adhered, they were subjected to various treatments. Subsequently, the cells were incubated with 5 µM JC‐1 (Beyotime, China) for 20 min at 37 °C in a light‐protected environment. After precooling, the cells were washed twice with the buffer provided in the kit. Changes in mitochondrial membrane potential were evaluated by observing the ratio of red to green fluorescence under a confocal microscope.

### Observation of Mitochondrial Ultrastructure

The NPCs were harvested using trypsin and then fixed with electron microscope fixative. The cells were subsequently fixed in 0.1 M phosphate buffer containing 1% osmium tetroxide. Following this, the cells were dehydrated through a graded series of ethanol concentrations and embedded for sectioning. Ultrathin sections with a thickness of 60–80 nm were prepared using an ultramicrotome. These sections were then stained with dicumyl acetate and lead citrate for enhanced contrast and examined under a transmission electron microscope (TEM, HITACHI HT7800, Japan).

### Assessment of Anti‐Inflammatory Activity

To assess the expression levels of inflammatory factors (TNF‐α, IL‐6, IL‐1β) using real‐time quantitative PCR (qPCR), NPCs were seeded into 12‐well plates and cultured to the logarithmic growth phase. Cells were treated with 50 µM TBHP for 12 h to induce inflammation. After TBHP treatment, cells were exposed to different experimental compounds or treatments for 48 h. Total RNA was extracted using TRIzol reagent, and the RNA concentration and purity were assessed using Nanodrop or agarose gel electrophoresis. The RNA was then reverse transcribed into cDNA using a reverse transcription kit. The qPCR was performed with SYBR Green PCR Master Mix and specific primers targeting TNF‐α, IL‐6, and IL‐1β, as well as a housekeeping gene β‐actin. The qPCR amplification was carried out in a thermal cycler with an initial denaturation at 95 °C for 3–5 min, followed by 35–40 cycles of 95 °C for 10 s, 60 °C for 30 s, and a final dissociation step to generate the melting curve. The relative gene expression was calculated using the 2^−ΔΔCT^ method. The primer sequences were shown in Table S1 (Supporting Information).

### Assessment of Anti‐Apoptotic Activity

The NPCs were seeded at a density of 1 × 10⁵ cells per well in a 24‐well plate and cultured until they reach the logarithmic growth phase. To establish an apoptosis model, the cells were treated with 50 µM TBHP for 24 h. Following this treatment, the culture medium containing TBHP was discarded. Subsequently, cells were exposed to various experimental treatments for an additional 2 days. After 2 days incubation, apoptosis was assessed using the TUNEL assay kit (Beyotime). In accordance with the manufacturer's instructions, the cells were first fixed and permeabilized, and then 50 µl of the TUNEL reaction mixture was added to each well. The cells were then incubated at 37 °C in the dark for 60 min. During this incubation, the TdT enzyme and labeled dUTP in the reaction mixture bind to the 3′‐OH ends of DNA strand breaks, resulting in fluorescent labeling. After incubation, the cells were washed three times with PBS to remove any unbound reagents. Finally, the cells were examined under a fluorescence microscope. Fluorescent signals observed in TUNEL‐positive cells reflect DNA fragmentation, a hallmark of apoptosis. A lower proportion of TUNEL‐positive cells indicates a stronger anti‐apoptotic effect.

To further validate the apoptotic status of NPCs following TBHP pre‐treatment, the Annexin V‐FITC Apoptosis Detection Kit (Beyotime, China) was employed. NPCs pre‐treated with TBHP and gels containing the solution were cultured in six‐well plates for 3 days. Apoptotic cells were identified through Annexin V‐FITC staining, and the extent of apoptosis was subsequently quantified by flow cytometry analysis.

### Senescence Assessment

To assess the anti‐aging activity of different treatments, SA‐β‐galactosidase (SA‐β‐gal) activity was evaluated using the SA‐β‐gal staining kit (Beyotime) according to the instructions. First, NPCs were seeded at a density of 1 × 10⁵ cells per well in a 24‐well plate and cultured to the logarithmic growth phase. Then, the cells were treated with 50 µM TBHP for 24 h to induce an aging model. After treatment, the culture medium containing TBHP was removed. Next, different experimental drugs were applied for intervention, and cells were co‐cultured for 2 days. After 2 days, the cells were stained using the SA‐β‐gal staining kit. SA‐β‐gal‐positive cells will appear blue under the optical microscope, indicating aged NPCs. Finally, SA‐β‐gal activity was quantified by calculating the percentage of SA‐β‐gal‐positive cells out of the total cells, with a higher percentage indicating a stronger anti‐aging effect.

In addition to SA‐β‐gal staining, immunofluorescence staining for p16 and p21 was conducted to further assess their roles in anti‐aging effects. NPCs were fixed with 4% paraformaldehyde and permeabilized with 0.1% Triton X‐100. Primary antibodies against p16 and p21 were incubated overnight at 4 °C, followed by incubation with fluorescently labeled secondary antibodies. The NPCs were counterstained with DAPI. Fluorescence signals were detected using confocal microscopy, and the expression levels of p16 and p21 were quantified by analyzing the intensity of the fluorescence signals.

### Regulatory Effects on ECM Synthesis

To evaluate the effects of different interventions on the ECM, NPCs were first seeded into 12‐well plates and cultured until they reached the logarithmic growth phase. The cells were then treated with 50 µM TBHP for 24 h to induce an oxidative stress and aging model, followed by treatment with various experimental drugs for 2 days.

First, NPCs samples were collected, and total RNA was extracted using TRIzol reagent. The RNA was then reverse‐transcribed into cDNA using a reverse transcription kit. Subsequently, qPCR was performed to measure the gene expression levels of Aggrecan, COL II, and MMP‐13. β‐actin was used as an internal control for data normalization, and the expression differences of the target genes in different experimental groups were analyzed. Concurrently, immunofluorescence staining was performed to assess the protein expression of COL II and MMP‐13. In brief, NPCs were fixed, permeabilized, and blocked, followed by incubation with specific primary antibodies. Afterward, fluorescently labeled secondary antibodies were used for staining. Finally, the expression of COL II and MMP‐13 was observed under a fluorescence microscope. The primer sequences were shown in Table S1 (Supporting Information).

In addition to qPCR and immunofluorescence analysis, WB analysis was used to evaluate the protein expression levels of Aggrecan, COL II, MMP‐13, and MMP‐3. First, treated cells were collected, and total protein was extracted using RIPA lysis buffer. Protein concentration was determined using the BCA method. Equal amounts of protein samples were separated by SDS‐PAGE and transferred to a PVDF membrane. The membrane was then blocked with BSA solution. Following this, specific primary antibodies against Aggrecan, COL II, MMP‐13, and MMP‐3 were added and incubated overnight at 4 °C. On the following day, the membrane was washed three times, and the appropriate HRP‐conjugated secondary antibodies were added for 1 h incubation at room temperature. After the incubation, electrochemiluminescence (ECL) was used to detect protein signals. The intensity of the target protein bands was analyzed to evaluate the protein expression levels of Aggrecan, COL II, MMP‐13, and MMP‐3 in the different experimental groups.

### Experimental Evaluation of IVDD in Rat Model

All animal procedures were conducted in compliance with the Technical Guidelines for the Care and Use of Laboratory Animals in China and were approved by the Experimental Animal Center at Zhengzhou University (ZZU‐LAC20250418 **[14]**). A total of 30 male SD rats, weighing between 180 and 240 g, were randomly divided into five groups (n = 6 per group): normal group (no puncture, NC group), IVDD group (puncture + PBS), blank gel group (puncture + blank gel), exosome group (puncture + gel + NP‐Exo), and NP‐Exo combined with D+Q group (puncture + gel + NP‐Exo + D+Q). After disinfection and anesthesia, a 20 G needle was vertically inserted into the center of the Co7/8 IVD. To avoid errors caused by the needle tip, the needle was rotated 360° and held in place for 1 min. After model establishment, an equal volume of the corresponding materials was injected into the IVD according to the experimental groupings.

### Experimental Evaluation of IVDD in Goat Model

A total of 30 male Boer goats were randomly divided into five groups (6 animals per group): normal group (no puncture, NC group), IVDD group (puncture + PBS), blank gel group (puncture + blank gel), exosome group (puncture + gel + NP‐Exo), and NP‐Exo combined with D+Q group (puncture + gel + NP‐Exo + D+Q). After preparation, disinfection, and anesthesia, the L4‐5 IVD was exposed via a retroperitoneal approach. A 16G needle was inserted into the left anterolateral side of the annulus fibrosus to a depth of 10 mm, reaching the nucleus pulposus center (with an average annulus fibrosus thickness of 7.5 mm). To avoid any errors caused by the needle tip, the needle was rotated 360° and held in place for 1 min. After completing the model establishment, equal amounts of the corresponding materials were injected into the IVD according to the experimental groupings, followed by layer‐by‐layer suturing. Postoperatively, penicillin (1.6 million units) was intramuscularly injected daily for 3 days.

### Radiological Evaluation

For rats, X‐ray and MRI examinations of the coccygeal IVD were performed at 4 and 8 weeks postoperatively. After obtaining X‐ray images, the disc height index (DHI) was calculated using ImageJ software. DHI was used to assess changes in disc height and is one of the key indicators for measuring the degree of disc degeneration.^[^ ^41^ ^]^ The change in DHI is expressed as DHI% (DHI of the treatment group/DHI of the NC group). A lower DHI% value indicates more severe disc degeneration. For MRI results, ImageJ software was used to analyze changes in IVD signal intensity based on T2‐weighted images. Additionally, IVDs were classified according to the system from grade I to V.^[^ ^42^ ^]^ Imaging examinations in goats were performed in the same manner as in rats.

### Histological Analysis

At 4 and 8 weeks postoperatively, SD rats were euthanized for IVD sample collection; likewise, goat IVD tissues were harvested at 8 weeks postoperatively for subsequent histological analysis. The samples were fixed in 4% paraformaldehyde solution for 48 h and decalcified in 10% EDTA decalcifying solution for 4–6 weeks. The specimens were then dehydrated in ethanol, embedded in paraffin, and sectioned for histological staining (H&E and SO‐FG) and immunohistochemical staining (COL II and MMP‐13). Microscopic images were taken to examine the IVD cells and morphology. The histological scoring for normal IVD was 5 points, moderate degeneration discs were scored between 6–11 points, and severe degeneration lumbar discs were scored between 12–14 points, as described previously^[^ ^43^ ^]^ (Table S2, Supporting Information).

### Statistical Analysis

All data were presented as the mean ± standard deviation (SD) and represent results from three independent experiments. Prior to statistical analysis, normality tests were conducted on the data. Comparisons between two groups were performed using Student's *t*‐test, while one‐way analysis of variance (ANOVA) followed by Tukey's post‐hoc test was used for multiple group comparisons (GraphPad Prism 8.0). Statistical significance was defined as *p < 0.05 and **p < 0.01.

## Conflict of Interest

The authors declare no conflict of interest.

## Author Contributions

S.F.C. and H.H. contributed equally to this work and share first authorship. S.F.C., H.J.L., and L.J. performed study conceptualization; S.F.C., H.H., Y.H.Z., L.Y.L., Z.S.W., J.M.Z., and L.H. performed methodology; H.H., Y.H.Z., H.W.K., G.W.S., C.F.S., Z.K.L., and K.Y.M. performed formal analysis and investigation; H.J.L., L.J., and C.W.L. wrote‐reviewed and edited; H.J.L., L.J., and S.F.C. performed funding acquisition; H.W.K., G.W.S., C.F.S., and K.Y.M. provided resources; H.J.L., L.J., and C.W.L. performed supervision All of the authors have read and approved the final manuscript.

## Supporting information



Supporting Information

## Data Availability

The data that support the findings of this study are available on request from the corresponding author. The data are not publicly available due to privacy or ethical restrictions.
