# Supplementary material for: Anti‐Swelling Hydrogel Combined With Nucleus Pulposus Cell Exosomes and Senolytic Drugs Efficiently Repair Intervertebral Disc Degeneration
Source: Adv Sci (Weinh). 2025 Sep 16;12(45):e13645. doi: 10.1002/advs.202513645 (PMC12677665; doi:10.1002/advs.202513645)
Supplement: Supplementary file 1 — Supporting Information [file ADVS-12-e13645-s001.docx]

**Supporting information**

**Anti-Swelling Hydrogel Combined with Nucleus Pulposus Cell Exosomes and Senolytic Drugs Efficiently Repair Intervertebral Disc Degeneration**

**Experimental section**

- 1. **Determination of Optimal Concentration and Ratio of Quercetin and Dasatinib for Combined Treatment**

The effect of different concentrations of quercetin (Q) and dasatinib (D) on the viability of NPCs was assessed using the CCK-8 assay. The results indicated that quercetin, at concentrations below 2 μM, had a mild promoting effect on cell growth, while concentrations above 4 μM resulted in an inhibitory effect on cell proliferation (**Fig.S1A**). Dasatinib, on the other hand, did not exhibit any proliferative potential, and at concentrations greater than 10 nM, it showed a strong inhibitory effect (**Fig.S1B**).

Subsequently, we evaluated the anti-aging effects and extracellular matrix (ECM) regulation of quercetin and dasatinib using qPCR. The results revealed that 50 μM of TBHP significantly induced cellular senescence and reduced ECM synthesis in NPCs. When quercetin was used at 2 μM, the anti-aging effect was most pronounced, and ECM synthesis was notably enhanced, as evidenced by a decrease in p21 expression, an increase in COL2 expression, and a decrease in MMP13 expression (**Fig.S1C-E**). Similarly, dasatinib showed optimal effects at a concentration of 10 nM in all aspects **(Fig.S1F-H)**.

Importantly, as shown in **Fig.S1I-K**, the combination of quercetin (2 μM) and dasatinib (10 nM) led to a significant enhancement of NPCs anti-aging capabilities, with the combination group exhibiting substantially higher cell viability compared to any of the monotherapy groups. Further qPCR analysis of p21, COL Ⅱ, and MMP-13 expression revealed that the combination treatment markedly reduced p21 expression, while COL Ⅱ expression was significantly increased and MMP-13 expression significantly decreased.

These findings suggest that the combined use of quercetin and dasatinib effectively modulates the aging process of NPCs and regulates ECM synthesis, resulting in the most favorable therapeutic outcomes. Overall, the combination of quercetin (2 μM) and dasatinib (10 nM) showed the most significant effects in terms of anti-aging and ECM regulation, making it the most promising treatment concentration for subsequent experiments.


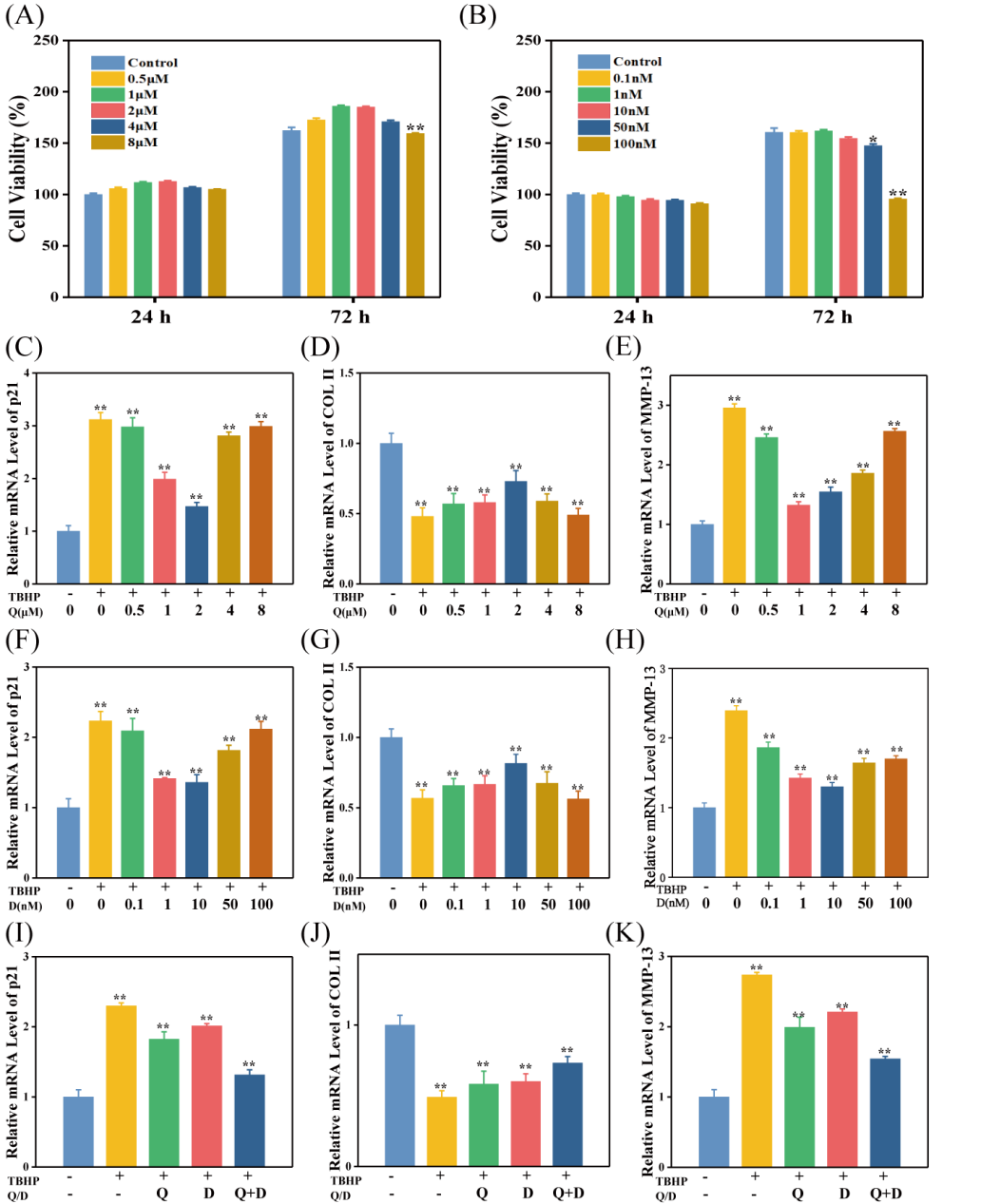


Figure S1. Determination of Optimal Concentration and Ratio of Quercetin (Q) and Dasatinib (D) for Combined Treatment. (A) Cell viability of rat NPCs after treatment with different concentration of Q. (B) Cell viability of rat NPCs after treatment with different concentration of D. (C-E) p21, COL II and MMP-13 expression of TBHP-treated cells incubated with Q of various concentrations. (F-H) p21, COL II and MMP-13 expression of TBHP-treated cells incubated with D of various concentrations. (I-K) Expression levels of p21, COL II, and MMP-13 in TBHP-treated cells incubated with Q, D, and their combination. Data are expressed as the mean ± SD (n=5).(*p< 0.05 vs. control group; **p< 0.01 vs. control group).

- 1. **Biocompatibility Evaluation**

The IVD is a tissue lacking blood vessels, which limits the local spread of the injected hydrogel and reduces its impact on other organs. Nevertheless, the potential toxic effects of the hydrogel on critical organs such as the heart, liver, spleen, lungs, and kidneys were still examined. In the eighth week of the study, tissue samples from these organs were collected from rats in various experimental groups. After fixation, histological evaluations were performed using H&E staining. The findings revealed that the structural integrity of each organ remained intact, with no signs of significant inflammation, cellular infiltration, apoptosis, or tissue injury. These results indicate that the hydrogel demonstrates exceptional systemic biocompatibility. As depicted in **Figure S2,** neither the hydrogel itself nor the drug-loaded variant induced notable adverse effects on the heart, liver, spleen, lungs, or kidneys. The alignment between in vivo and in vitro experimental outcomes further confirms the strong safety profile of the hydrogel.


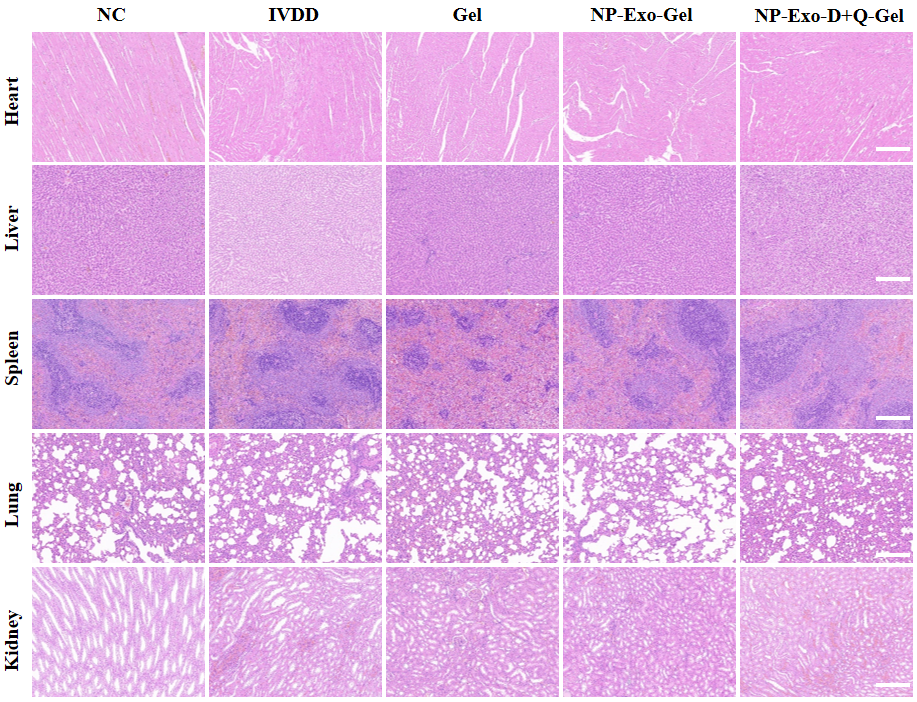


Figure S2**.** Biocompatibility evaluation. Images of H&E staining of hearts, livers, spleens, lungs, and kidneys from rats at 8 weeks (bar: 1 mm).

**
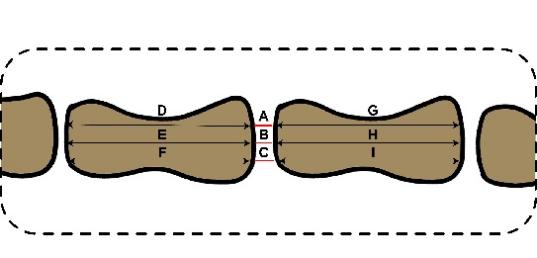
**
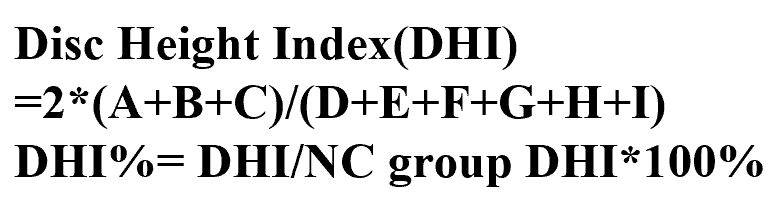


Figure S3. Calculation formula and diagram of DHI.

**Table S1. Primers used in real-time PCR**

| Gene | Primer | Sequence |
| --- | --- | --- |
| β-action | Forward  Reverse | AGATCAAGATCATTGCTCCTCCT  ACGCAGCTCAGTAACAGTC |
| TNF-a | Forward  Reverse | AATGGCATGGATCTCAAAGACAACC  GAGGCTGACTTTCTCCTGGTATGAA |
| IL-1β | Forward  Reverse | TTGAGTCTGCACAGTTCCCC  GTCCTGGGGAAGGCATTAGG |
| IL-6 | Forward  Reverse | TCTCCGCAAGAGACTTCCAG  AGCCTCCGACTTGTGAAGTG |
| Aggrecan | Forward  Reverse | AGGTCGTGGTGAAAGGTGTTGTG  TGGTGGAAGCCATCCTCGTAG |
| Collagen2 | Forward  Reverse | GCTCCCAGAACATCACCTACCA  ACAGTCTTGCCCCACTTACCG |
| MMP-13 | Forward  Reverse | CAAAGACTATCCCCGCCTCATAGAA  GGAATTTGTTGGCATGACTCTCACA |

**Table S2. Histological  scores  of  discs**

| **Histological  scores  of  disc** |
| --- |
| **Morphology of the nucleus pulposus** |
| **Score：** |
| 1. Round shape, occupying more than 50% of the disc area in midsagittal sections |
| 2. Round or irregular shape, occupying 25% to 50% of the disc area |
| 3. Clearly irregular shape, occupying less than 25% of the disc area |
| **Cellularity of the nucleus pulposus** |
| **Score：** |
| 1. Normal cellularity with stellate-shaped cells and uniform distribution of proteoglycan matrix throughout the nucleus |
| 2. Reduced cell number with the appearance of rounded cell morphology |
| 3. More than 50% reduction in cell number, with cells forming aggregates separated by dense regions of proteoglycan matrix |
| **Morphology of the annulus ﬁbrosus** |
| **Score：** |
| 1. Collagen lamellae are well-organized, with no evidence of wavy or serpentine fibers |
| 2. Disruption of annular fibers involves one-third or less of the annulus |
| 3. More than one-third of the annulus shows fiber rupture, accompanied by inward bulging of the annular structure |
| **Cellularity of the annulus ﬁbrosus** |
| **Score：** |
| 1. Fibroblasts account for more than 75% of the total cell population |
| 2. Neither fibroblasts nor chondrocytes comprise more than 75% of the total cells |
| 3. Chondrocytes account for more than 75% of the total cell population |
| **Endplates** |
| **Score:** |
| 1. Endplate structure is continuous and intact |
| 2. Endplate structure is disrupted or interrupted |
